# Functional Status of Peripheral Blood T-Cells in Ischemic Stroke Patients

**DOI:** 10.1371/journal.pone.0008718

**Published:** 2010-01-14

**Authors:** Antje Vogelgesang, Verena E. L. May, Uwe Grunwald, Maren Bakkeboe, Soenke Langner, Henry Wallaschofski, Christof Kessler, Barbara M. Bröker, Alexander Dressel

**Affiliations:** 1 Department of Neurology, University of Greifswald, Greifswald, Germany; 2 Institute for Immunology and Transfusion Medicine, University of Greifswald, Greifswald, Germany; 3 Department of Radiology, University of Greifswald, Greifswald, Germany; 4 Institute for Clinical Chemistry and Laboratory Medicine, University of Greifswald, Greifswald, Germany; New York University, United States of America

## Abstract

Stroke is a major cause of disability and leading cause of death in the northern hemisphere. Only recently it became evident that cerebral ischemia not only leads to brain tissue damage and subsequent local inflammation but also to a dramatic loss of peripheral blood T-cells with subsequent infections. However, only scarce information is available on the activation status of surviving T cells. This study therefore addressed the functional consequences of immunological changes induced by stroke in humans. For this purpose peripheral blood T-cells were isolated from 93 stroke patients and the expression of activation makers was determined. In addition ex vivo stimulation assays were applied to asses the functionality of T cells derived from blood of stroke patients. Compared to healthy controls, stroke patients demonstrated an enhanced surface expression of HLA-DR (p<0.0001) and CD25 (p = 0.02) on T cells, revealing that stroke leads to T cell activation, while CTLA-4 remained undetectable. In vitro studies revealed that catecholamines inhibit CTLA-4 upregulation in activated T cells. Ex vivo, T cells of stroke patients proliferated unimpaired and released increased amounts of the proinflammatory cytokine TNF-α (p<0.01) and IL-6 (p<0.05). Also, in sera of stroke patients HMGB1 concentrations were increased (p = 0.0002). The data demonstrate that surviving T cells in stroke patients remain fully functional and are primed towards a TH1 response, in addition we provide evidence that catecholamine mediated inhibition of CTLA-4 expression and serum HMGB1 release are possible mediators in stroke induced activation of T cells.

## Introduction

Stroke is a major cause of disability and leading cause of death in the northern hemisphere, second only to cardiac disease, and is accompanied by a grave economic impact [1]. The clinical course of stroke patients is not solely determined by the extent of brain damage and the resulting neurological deficit, but often complicated by post stroke infections. These affect between 10% and 35% of patients in the week following stroke [2], [3]. Only recently it became evident that cerebral ischemia not only leads to brain tissue damage and subsequent local inflammation but also to dramatic alterations of the peripheral immune system. First reported in a mouse model of stroke [4], we and others have demonstrated that also in humans stroke induces an immediate loss of lymphocytes. The rapid lymphocytopenia, predominantly seen among CD4^+^ T-cells, is attributed to apoptosis due to increased cortisol and catecholamine release. A delayed recovery of the CD4^+^ T-cell counts indicates an increased risk for subsequent infection. In addition to cellular parameters, serum cytokine concentrations have also been shown to differ between patients with and without subsequent infections. Of interest, IL-10 a cytokine with strong anti-inflammatory properties and IL-6 known for its proinflammatory capacities are both elevated in stroke patients with subsequent infection [5], [6], [7].

To better understand the role of soluble modulators in stroke induced immunosuppression we studied prototypical pro- and anti-inflammatory cytokines and determined HMGB1 levels. HMGB1 is released into serum either by secretion from activated monocytes, T cells, thrombocytes or endothelial cells or by passive release from necrotic tissue [8], [9], [10]. Serum HMGB1 strongly promotes inflammation, regulates dendritic cell function and migration, skews the T cell response towards a Th1 profile and induces proliferation and the release of IL-6 [11], [12], [13] Consequently, HMGB1, which has been shown to be elevated within 24 hours following stroke or myocardial infarction, could act as important player in the modulation of post stroke immune responses. Furthermore, this molecule has been shown to contribute to brain tissue destruction following brain injury [13], [14], [15]. However, to determine the biological relevance of elevated serum HMGB1 the concentrations of its functional inhibitors the soluble and the endogenous secretory receptor for advanced glycation end products (sRage and esRage) need to be determined [16].

The more recent research on immunological sequelae of ischemic stroke has delineated mechanisms that result in rapid alterations of the immune system and identified several immunological factors associated with subsequent infection. Why these alterations would increase the susceptibility to bacterial infections is largely unexplored. This study therefore addressed the functional consequences of immunological changes induced by stroke in humans. Of special interest were the kinetics of serum cytokine concentrations and differences in T cell function between stroke patients with subsequent infection and those, who remained free from this complication.

## Materials and Methods

Patients with onset of stroke symptoms less than 12 hours before admission to the stroke unit and without clinical signs of infection on admission were eligible for recruitment into the study. Ischemic stroke was diagnosed clinically and by cerebral CT. Routine cerebral CT images were acquired on a 16-row multislice CT scanner (Somatom 16; SIEMENS Medical Systems). To calculate lesion size, images were analyzed using OSIRIX 2.5.1.

Blood was obtained immediately on admission and between 6:00 a.m. and 7:00 a.m. on days 1, 7 and 14 thereafter. A previous publication included data from 46 of the 93 patients and 14 of the 17 controls analysed in the current study [7]. Patient details are summarized in Table 1.

**Table 1:** 

	Total No.	Age Mean (Range)	NIHSS Median (Range)	Lesion Volume (mm^3^) Median (Range)	Male	Female
**Nonstroke control subjects** 1)	17	65	NA	NA	6	8
		(50–94)				
**Total patients included**	93	69.8	15	141120	47	46
		(41–92)	(2–26)	(441–2 954 304)		
**Infected cohort^2)3)^**	17	70.6	17	162180	10	7
		(49–92)	(2–26)	(10230–2 954 304)		
**Noninfected cohort** 3)	24	63.2	9	64638	14	10
		(43–88)	(2–20)	(441–2 551 298)		
**Infection status not set** 3)	52	72.5	17	131585	23	29
		(41–89)	(3–26)	(689–617792)		

1) Nonstroke control subjects were healthy individuals (n = 12) or had other diseases (Parkinson's disease n = 1, Bell's palsy n = 1, hypertensive crisis n = 1, diabetes mellitus n = 1,or polyneuropathy n = 1).

2) Infected cohort patients suffered from: 14 pneumonia n = 14, ,fever of unknown origin n = 1, erysipelas n = 1, intravenous catheter abscess n = 1. 3 patients additionally aquired urinary tract infection.

3) Patients were assigned as described in material and methods section.

NA: not applicable.

### Ethics Statement

The study protocol was approved by the ethics committee of the Medical Faculty, University of Greifswald (No. III UV 30/01). All patients gave fully written informed consent directly or through a surrogate where appropriate.

### Definition of Infection

For the purpose of this study, we applied the following criteria to define infection: (1) presence of clinical signs of infection (pneumonia, urinary tract infections, fever of unknown origin); (2) serum concentrations of C- reactive protein >50 mg/mL; and (3) procalcitonin serum concentrations >0.5 ng/mL. To compare patients who developed infection after stroke with those who did not, 2 cohorts were formed. In the infected cohort, all 3 criteria for infection had to be fulfilled on day 7 or 14. In the noninfected cohort, none of the criteria was matched throughout the whole study period. Patients who matched some criteria but not all were not assigned to either cohort. Patients with signs of infection on admission were not recruited into the study as published previously [7].

Peripheral blood mononuclear cells (PBMC) were isolated by standard ficoll gradient centrifugation. Cell culture was performed at a cell density of 2×10^5^ cells/well in 96 well u-bottom plates (Nunc, Roskilde, Denmark) in 200 µl RPMI supplemented with 1% glutamine, 1% penicillin, 1% streptomycin and 10% human AB serum. For the cytokine and proliferation assays cells were incubated with 1 µg/ml PHA (Abbot, Wiesbaden, Germany) for 72 h. Cell culture supernatants were collected after 48 h of incubation.

IFN-γ, IL-1β, IL-4, IL-5, IL-6, IL-8, IL-10, IL-12p70, TNF-α and TNF-β were measured using a Human Th1/Th2 multiplex kit (Bender MedSystems, Vienna, Austria). The cytokines were quantified by FACS analysis on a Becton Dickinson FACScan. Data were evaluated using the FlowCytomix Pro 2.1 software package provided by Bender MedSystems. IL-17 was detected by ELISA (eBioscience, Frankfurt, Germany) following the manufacturer's protocol as was HMGB1 (IBL, Hamburg, Germany).

Cortisol and metanephrine yield was determined in serum samples after storage at −80°C by ELISA according to the manufacturer's recommendations (Cortisol or MetCombi plasma ELISA®; IBL, Hamburg, Germany).

CTLA-4 expression was determined on peripheral blood T cells from human stroke patients and healthy age-matched controls by immune fluorescence staining. Cytospins of PBMC were prepared, acetone-fixed, and Fcγ-receptors were blocked with human IgG (Cohn fraction II, Sigma, Deisenhofen, Germany). Slides were incubated with anti-human CD3 and biotinylated anti-human CTLA-4 monoclonal antibodies (BD Biosciences, USA) and then post-fixed with 4% PFA (Sigma, Deisenhofen, Germany). Endogenous peroxydase was inactivated with 3% H_2_O_2_ (Merck, Darmstadt, Germany). After incubation with Streptavidine-POD (1∶500 in PBS) and biotinyltyramid (1∶200 in amplification diluent) for signal amplification (TSA™ Biotin System, NEN Life Science Products, Boston, MA, USA), the cytospins were finally incubated with goat anti–mouse IgG1-FITC (Southern Biotechnology Associates,Birmingham, AL, USA) and streptavidine-TRITC for visualization (Jackson Immuno Research, Dianova, West Grove, PA, USA). Microscopic quantification was performed by observers, who were blinded for the identity and grouping of the patient. A minimum of 500 cells were evaluated per sample.

Hormone effects on PHA stimulated PBMC were assessed in 48 h cultures in the presence of 1×10^−7^ M epinephrine or norepinephrine or 1×10^−6^ M dexamethasone (all Sigma, Deisenhofen, Germany) and the appropriate control conditions.

Proliferation was measured by standard ^3^H-thymidin incorporation assays. ^3^H-thymidin (Amersham, Braunschweig, Germany) was added to the cell culture after 48 h. Its incorporation was determined on a phosphorimager (Molecular Dynamics, Sunnyvale, CA, USA) after additional incubation of 18 h. Proliferation is shown as stimulation index (SI) calculated as counts per minute (CPM) with stimulants/CPM without stimulants. Cytokine concentrations of supernatants are given as the differences between PHA-stimulated and unstimulated values.

### FACS Analysis of Helper Cell Activation

Lymphocyte subpopulations were estimated on a FACSCalibur (Becton Dickinson, Heidelberg, Germany). Aliquots of 100 µl of whole blood, anticoagulated with EDTA, were incubated with appropriate combinations of fluorescence-conjugated monoclonal antibodies. After lysing of erythrocytes (FACS Lysing solution, BD Biosciences, Heidelberg, Germany) cells were washed once (PBS, 1% FCS, 0.2% NaN3) before measurement. Monoclonal antibodies used to determine expression of cell surface molecules were CD25-FITC (DAKO Cytomation, Hamburg, Germany), CD4-PerCP or CD4-PE, CD3-PerCP, and HLA-DR-FITC (all BD Biosciences, Heidelberg, Germany). Absolute number of lymphocytes used for calculation of absolute counts of lymphocyte subpopulations were estimated in a separate Trucount tube stained with anti-CD45-PerCP mAb (BD Biosciences, Heidelberg, Germany) measured without washing after lyses of erythrocytes.

### Statistical Analysis

All data sets were tested for deviations from Gaussian distribution using the Kolmogorov-Smirnov test. Data that passed the test were analyzed using repeated-measures ANOVA and Bonferroni's multiple comparison test as post test. Data failing the normality test were analyzed using the Friedmann test with Dunn's multiple comparison test as post test. The comparison of two sets of data was performed using paired or unpaired student's t-test, as appropriate for normally distributed data and Kruskal Wallis test for those without Gaussian distribution. All analyses were carried out using the software GraphPad-PRISM 5.0 (GraphPad Software inc., San Diego, CA, USA). A p<0.05 was regarded as significant.

## Results

### Th1-Type Cytokines Are Downregulated in Sera of Patients with Acute Stroke

**Figure 1:**
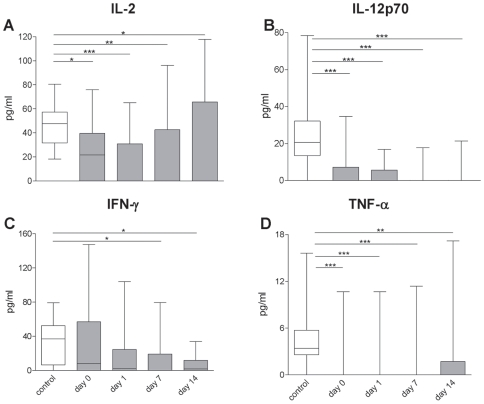
Th1-type cytokines are downregulated in stroke patients. IL-2, IL-12p70, IFN-γ and TNF-α serum cytokine levels are significantly reduced in sera of stroke patients (grey bars) compared to healthy controls (white bars) in the first two weeks following stroke. * p<0.05; ** p<0.01; *** p<0.001. Boxes with medians and whiskers min to max. n_(IL-2, IL-12p70, TNF-α)_ = 16; 20; 20; 21; 13; n_(IFN-γ)_ = 16; 20; 20; 19; 12 (control; day 0; day 1; day 7; day14).

To characterize the effects of ischemic stroke on the cytokine milieu in the serum pro- and anti-inflammatory cytokines in stroke patients and control subjects were determined. Serum concentrations of the proinflammatory cytokines TNF-α (p = 0.0001), IL-12p70 (p<0.0001), IL-2 (p = 0.0016) and IFN-γ (p<0.05) were significantly decreased (Fig. 1). IL-4 and IL-8 serum concentrations were not altered, while IL-1β, IL-5, and TNF-β remained below the threshold of detection in the majority of patients and controls (data not shown).

### Serum HMGB1 Is Upregulated in Stroke Patients

**Figure 2:**
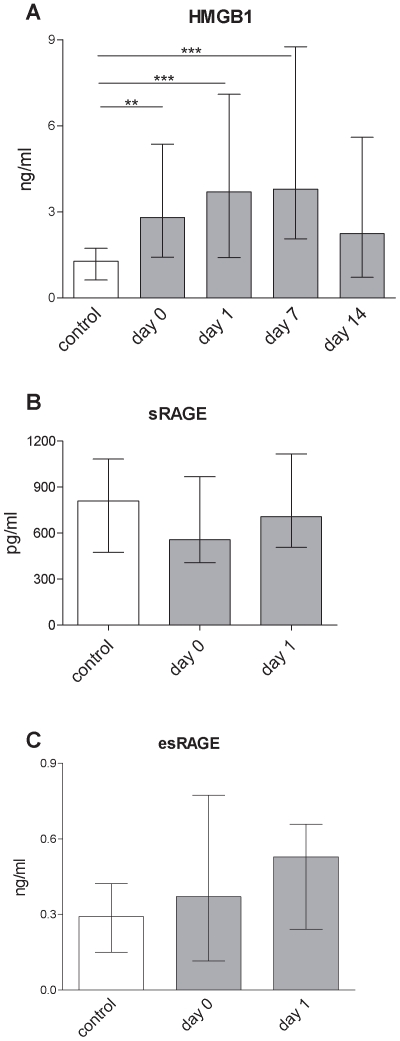
HMGB1 is upregulated in serum of stroke patients. HMGB1 is upregulated in serum of stroke patients (grey bars) compared to healthy controls (white bars) in the first two weeks following stroke while its decoy receptors sRAGE and esRAGE remain indistinguishable within 48h following stroke. ** p<0.01; *** p<0.001. Medians and interquartile ranges. n_(HMGB1)_ = 16; 87; 84; 46; 24; n_(sRAGE,)_ 12; 32; 29, _esRAGE)_ = 10; 37; 34, (control; day 0; day 1; day 7; day14).

HMGB1 was rapidly upregulated following stroke and elevated serum levels persisted throughout the study period of 14 days (p = 0.0002). Importantly, the serum concentrations of the natural inhibitors of HMGB1, sRAGE and esRAGE, did not change (Fig. 2).

Since HMGB1 has been shown to induce IL-6 we tested relations between infarction size, IL-6 and HMGB1. While HMGB1 serum levels significantly correlated with IL-6 (on day 7: r = 0.4896, p = 0.0152), this was not the case for brain tissue destruction as assessed by CT morphometry (data not shown).

### CD4^+^ T-Cells in the Peripheral Blood of Stroke Patients Are Activated

**Figure 3:**
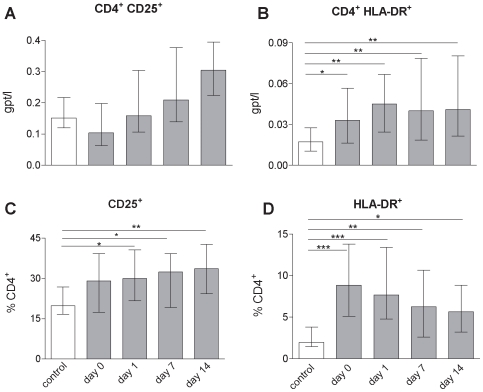
CD4^+^ T-cells in the peripheral blood of stroke patients are activated. Comparison of expression of activation markers on lymphocytes between healthy controls (white bars) and stroke patients (grey bars) in the first two weeks following stroke reveal that CD4^+^ T-cells in the peripheral blood are activated. * p<0.05; ** p<0.01; *** p<0.001. Medians and interquartile ranges. n_(CD4_^+^_CD25_^+^_, CD25_^+^_%CD4_^+^_)_ = 14; 33; 32; 24; 14; n_(CD4_^+^_HLA-DR_^+^_)_ = 12; 64; 66; 42; 22; n_(HLA-DR_^+^_%CD4_^+^_)_ = 12; 63; 66; 43; 22; (control; day 0; day 1; day 7; day14).

It is well documented that following stroke a large fraction of T cells is rapidly lost from the peripheral blood. To determine the activation status of the remaining T cells the expression of the established T cell activation markers IL-2 receptor (CD25) and HLA-DR were measured. Compared to controls, an increased proportion of CD4^+^ T-cells expressed CD25 (p = 0.02) or HLA-DR (p<0.0001), respectively (Fig. 3). This corresponded to a net increase in CD4^+^ HLA-DR^+^ T-cell numbers (p = 0.0055) despite the reduction of the total numbers of CD4^+^ T-cells. A similar trend was observed for CD4^+^CD25^+^ T-cells. This shows that in stroke CD4^+^ T-cells in the peripheral blood become activated.

**Figure 4:**
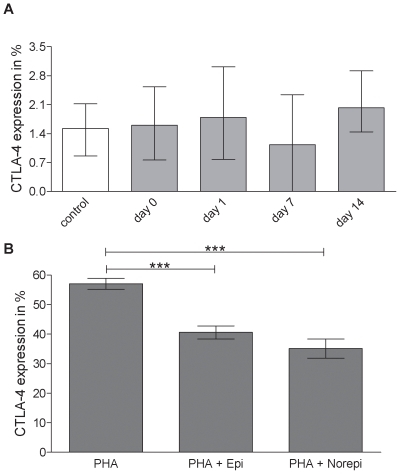
Lack of CTLA-4 expression and inhibition by catecholamines. A) The percentage of PBMC expressing CTLA-4 is undistinguishable between healthy controls (white bars) and stroke patients (grey bars) in the first two weeks following stroke. Medians and interquartile ranges. n = 9; 31; 33; 27; 20; (control; day 0; day 1; day 7; day14). B) Percentage of CTLA-4 expression on PBMC decreases after *in vitro* treatment with PHA+epinephrine or PHA+norepinephrine compared to treatment with PHA alone. *** p<0.001. Medians and interquartile ranges. .n = 13; 6; 9; (PHA; PHA+Epi; PHA+Norepi).

CTLA-4, which is constitutively expressed on Treg cells but is also up-regulated by activation of T effector cells, was determined by immunoflourescence staining. Interestingly no CTLA-4 upregulation was observed (Fig. 4A).

### Catecholamines Inhibit CTLA-4 Expression on Activated T-Cells

We hypothesized that stroke-induced catecholamines and steroids could account for the lack of CTLA-4 upregulation and examined the influence of these hormones on CTLA-4 expression *in vitro* (Fig. 4B). Stimulation with the mitogen PHA induced strong upregulation of CTLA-4 on CD4^+^ T-cells, which was counteracted by the addition of catecholamines (p<0.0001; Fig. 4) but not by dexamethasone into the cell culture (data not shown).The concentrations of dexamethasone, epinephrine and norepinephrine used in these experiments did not enhance apoptosis, which excludes cell death as a reason for the catecholamine-mediated changes in CTLA-4 expression (data not shown). The data suggest that the high concentrations of catecholamines in acute stroke patients could explain why CTLA-4 was not upregulated on activated CD4^+^ T-cells.

### Surviving T Lymphocytes Are Functional

**Figure 5:**
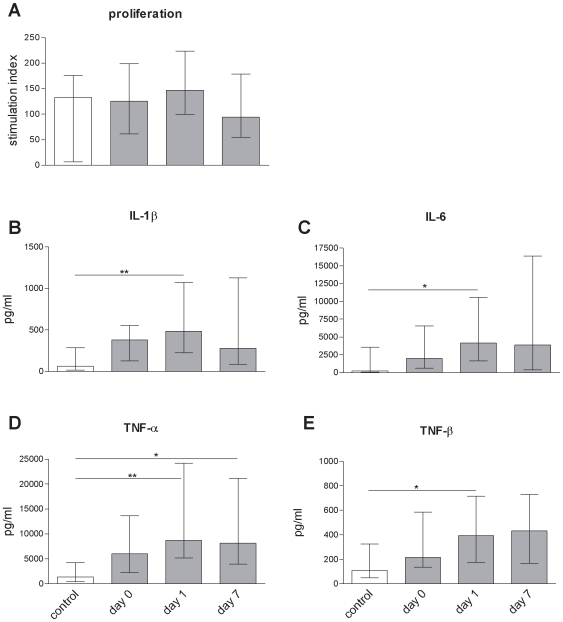
Surviving T lymphocytes are functional. A) The proliferation expressed as stimulation index of T cells after *ex vivo* stimulation with PHA reveals normal proliferative responses of stroke patients (grey bars) lymphocytes compared to healthy controls (white bars) in the first week following stroke. Medians and interquartile ranges. n = 7; 36; 35; 32; (control; day 0; day 1; day 7). B–E) E*x* *vivo* stimulation of T cells with PHA leads to increased proinflammatory cytokine release in supernatants of stroke patients cells (grey bars) compared to healthy controls (white bars) within the first week following stroke. * p<0.05; ** p<0.01. Medians and interquartile ranges. n_(IL-1β, IL-6, TNFα, TNF-β)_ = 10; 37; 36; 28; (control; day 0; day 1; day 7).

To assess whether the surviving lymphocytes in stroke patients retained their functional capacity, *ex vivo* stimulation assays were performed. The proliferative capacity of the PBMC was unaltered during the first week following ischemic stroke (Fig. 5 A). The cells were stimulated with PHA and cytokine release was measured. This revealed a predominance of the proinflammatory cytokines IL-6, IL-1ß, TNF-α and TNF-β (Fig. 5 B–E) while IL-17, HMGB1 and IL-4, IL-5, IL-8, IL-10, IL-12p70 and IFN-γ remained unchanged (data not shown). Thus, the function of the surviving lymphocytes was intact and skewed towards proinflammation.

### Differences between Stroke Patients with and without Subsequent Infection

**Figure 6:**
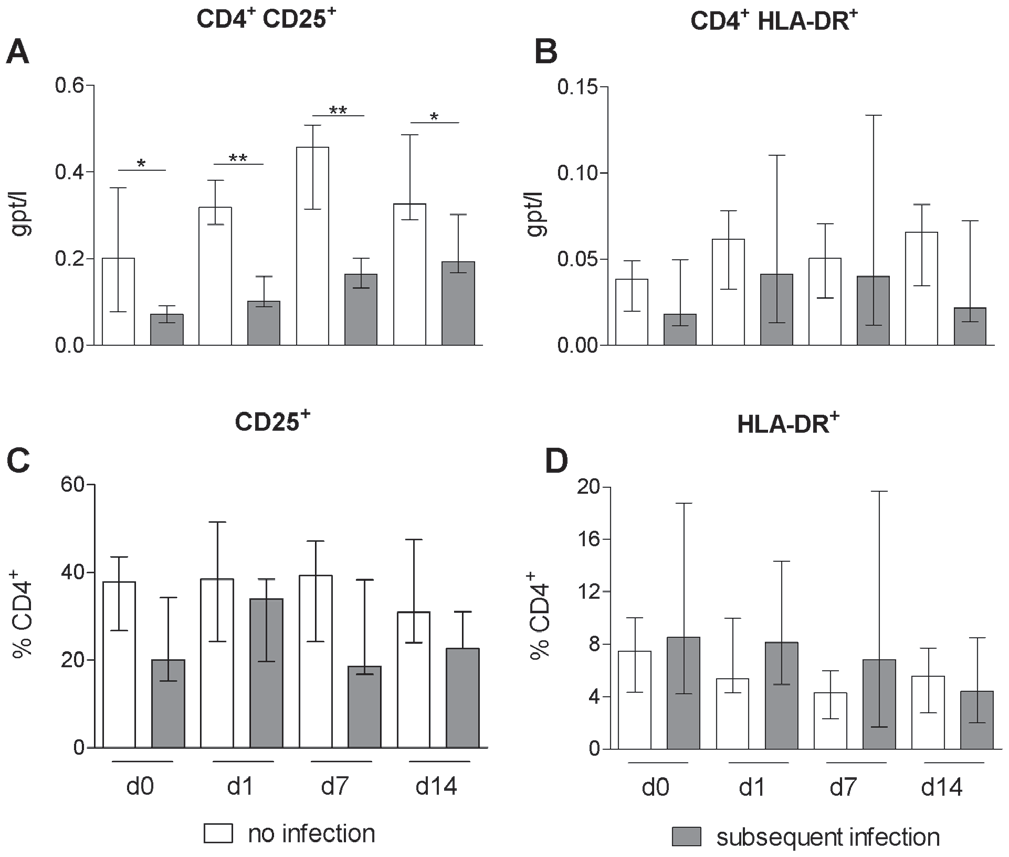
Relative proportion of activated T cells were undistinguishable between patient subgroups. Activation markers on lymphocytes from stroke patients without subsequent infection (white bars) remained undistinguishable from those of stroke patients with subsequent infection (grey bars) within the relative proportion of lymphocytes in the first two weeks following stroke. * p<0.05; ** p<0.01. Medians and interquartile ranges. . n_(CD4_^+^_CD25_^+^_, CD25_^+^_%CD4_^+^_)_ = 5; 7; 7; 7; 7; 6; 6; 3; n_(CD4_^+^_HLA-DR_^+^_, HLA-DR_^+^_%CD4_^+^_)_ = 14; 12; 16; 11; 12; 9; 8; 5 (no infection day 0; subsequent infection day 0; no infection day 1; subsequent infection day 1; no infection day 7; subsequent infection day 7; no infection day 14; subsequent infection day 14).

We have previously reported that a delayed recovery of CD4^+^ T-cell counts predisposes to a subsequent infection. In agreement with this finding absolute numbers of CD4^+^CD25^+^ T-cell were higher in the non-infected cohort (p<0.05;). while the the relative proportion of activated T cells did not differ (Fig. 6). Also, comparison of cytokine patterns following ex vivo stimulation of T cells from the infected and the non-infected cohort revealed no differences (data not shown).

**Figure 7:**
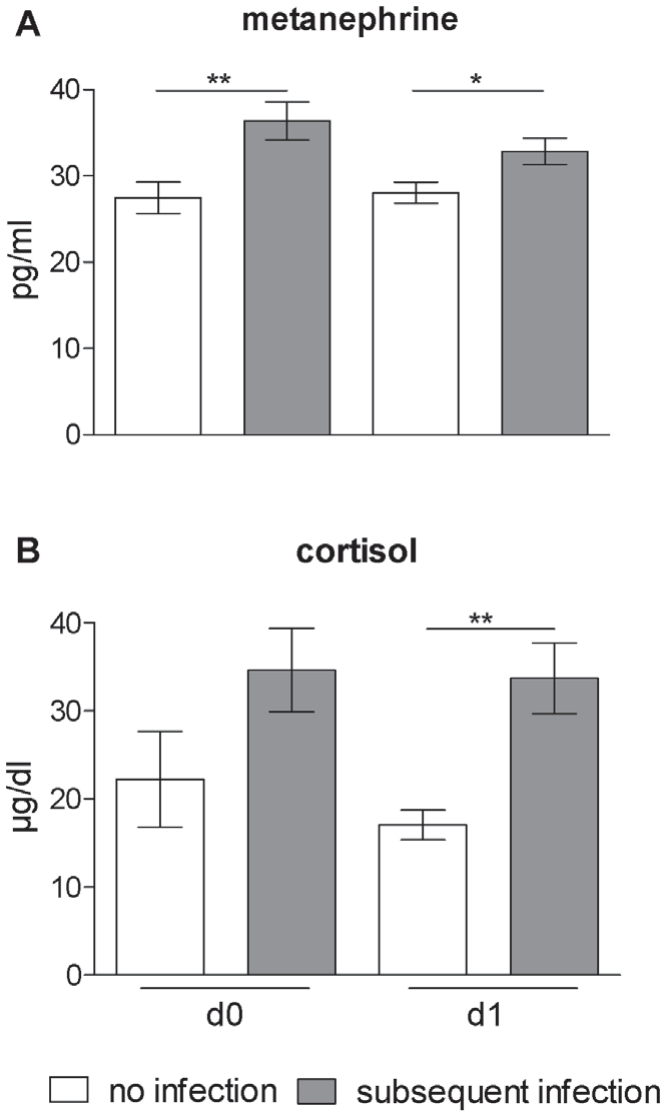
Increased metanephrine and cortisol serum levels in patients with subsequent infection. Metanephrine and cortisol serum levels are increased in serum of stroke patients with subsequent infection (grey bars) compared to stroke patients without subsequent infection (white bars) within 24h following stroke. * p<0.05; ** p<0.01. Means±SEM. n_(Metanephrine)_ = 8; 8; 8; 7; n_(Cortisol)_ = 10; 8; 9; 9; (no infection day 0; subsequent infection day 0; no infection day 1; subsequent infection day 1).

Serum concentrations of steroids and metanephrine were increased in patients that went on to develop infection, further supporting a role of these hormones in the immunosuppression following stroke (Fig. 7). In addition, serum IL-6 levels and IL-10 levels were significantly increased in the subgroup with subsequent infection as reported previously [7] and confirmed within this cohort (data not shown). However, this was not paralleled by differences between the two patient groups for other proinflammatory serum cytokine or HMGB1 concentrations (data not shown).

## Discussion

Previous studies have addressed the changes in serum cytokines and cellularity following ischemic stroke in humans [5], [6], [7], [17], [18], [19]. This study expands the recent findings and addresses the activation status and the ex vivo functionality of surviving T-lymphocytes from peripheral blood from post- stroke patients, for only little information has been available on this aspect.

In comparison to healthy controls; stroke patients demonstrated an enhanced surface expression of HLA-DR and CD25 on T cells, revealing that stroke leads to T cell activation. Furthermore ex vivo stimulation assays demonstrate normal proliferative responses and enhanced secretion of proinflammatory cytokines, which argues for Th1 priming in stroke patients. The apparent contradiction of intact T cell responses and decreased proinflammatory serum cytokines can be reconciled when the pronounced T cell loss in stroke is taken into account. This explanation is supported by a report by Haeusler et al who have reported lower TNF-α secretion when stimulating defined volumes of whole blood ex vivo [20]. Also stroke has been shown to result in decreased proinflammatory cytokine secretion by other cell types such as macrophages resulting in the net-decrease of serum cytokines despite enhanced T-cell activation [6].

Urra et al have reported that a reduced proportion of CD4+ CD3+ T-cells contain TNF-α when stimulated for 4hrs with PMA-Ionomycin [19]. This difference may be explained by the mitogens used in the studies: PMA-Iononmycin but not PHA has been shown to rapidly downregulate CD4 expression on T-cells [21]. Therefore PMA-Ionomycin activated CD4+ T-cells producing TNF-α may be missed when combined with FACS analysis gating on CD4+ T-cells.

CTLA-4, a tightly regulated T cell surface molecule, is upregulated following activation of both, Treg and T effector cells [22]. CTLA-4 has an important inhibitory function in T cell regulation, and is assumed to mediate immunosuppressive properties of human Foxp3^+^ Treg cells [23]. In this study, CTLA-4 surface expression remained at the level of healthy age-matched individuals even though other surface markers of T-cell activation were upregulated.

Our in vitro data suggest that the catecholamine release following stroke is causally related with the inhibition of CTLA-4 induction in activated T cells. While the concentrations of catecholamines used for these in vitro experiments exceed serum levels they did not induce T-cell apoptosis. We conclude that the catecholamine concentrations chosen for the in vitro experiments are below the concentrations occurring in vivo where catecholamine induced apoptosis has been observed [4]. The catecholamine mediated inhibition of CTLA-4 expression in activated T cells might reflect one of the key events leading to poststroke activatability and Th1 priming of T cells.

While the pathophysiology of stroke induced lymphocyte loss is increasingly understood, the mechanisms resulting in T cell activation are largely unknown. HMGB1 is known to induce proliferation and Th1 priming of T-lymphocytes and is released into serum by activated epithelium and by tissue undergoing necrosis. HMGB1 is therefore a candidate molecule that could serve as possible mediator of T cell activation in stroke. Indeed, in a small series of stroke patients serum HMGB1 was elevated as demonstrated in western blots. The natural inhibitors of HMGB1 sRage and esRage were not determined, though [13]. Here we confirm and extend this previous observation since we also detected increased serum concentrations of HMGB1 by ELISA in a larger cohort of stroke patients. Importantly, this was not paralleled by an increase in sRage and esRage, which are known to inhibit HMGB1. Of note, the HMGB1 serum kinetics resemble closely the kinetics observed for the absolute numbers of CD4^+^ T-cells expressing HLA-DR in this study. In addition, HMGB1 correlated with IL-6 serum concentration, a cytokine that is readily induced by HMGB1. Together, these observations support the hypothesis that HMGB1 serves as a link between brain tissue destruction and peripheral activation and Th1 priming of T-lymphocytes.

Stroke induced immunosuppression that appears causally related to increased infection rates in this patient population has been described over the last six years in animal models and human stroke patients. There is converging evidence suggesting that a hormone storm of catecholamines and steroids immediately after cerebral ischemia results in T cell apoptosis and reduced MHC expression on monocytes [4], [6], [19]. The data reported here also confirm the linkage of hormone concentrations with subsequent infection [5], [6], [24]. In addition, we and others have described several immunological markers including CD4^+^ T-cell counts, serum IL-6 levels, and TNF-α release by macrophages that are associated with the occurrence of infection in stroke patients [5], [6], [7], [20]. While the occurrence of pneumonia in stroke patients is commonly considered to be a strong independent predictor of clinical outcome this finding has recently been disputed [6], [25], [26]. These differences may be related to the variability of definitions used to define infected and non-infected patients. In this study we used the identical stringent definitions of post stroke infection and non-infected patients reported previously; as a consequence, all parameters obtained on admission and on day 1 were determined in the absence of clinical and paraclinical signs of infection. This rigorous approach has previously identified CD4^+^ T-cell counts, and serum IL-6 concentrations as independent predictors of infection [7].

In the current study, we demonstrate that the surviving T-cells in the peripheral blood are activated and secrete proinflammatory cytokines. These data could be interpreted as evidence supporting the hypothesis that susceptibility to bacterial infections is a quantitative effect of low CD4^+^ T-cell counts. Alternatively, low CD4^+^ T-cell counts might represent a surrogate marker that is closely related to the magnitude of multiple immunosuppressive effects induced by cerebral ischemia. The challenge will now be to identify the causative immune suppressive mechanisms.

In summary, here we provide a detailed analysis of T cell functionality in stroke patients. We demonstrate a rapid activation of T cells that are primed towards a Th1 response. This was true for both patient subgroups; those with and those without subsequent infection.

Our data point towards two mechanisms which may mediate the activation of T cells in stroke patients: while increased HMGB1serum levels may activate surviving lymphocytes in stroke patients a downregulatory mechanism, the expression of CTLA-4 remains suppressed.

Further studies are needed to characterize the immunosuppressive cascades triggered by cerebral ischemia. Investigators should take into account that immune suppression may also have beneficial effects by preventing secondary autoimmune disorders. To which degree the stroke induced alterations of the immune system that apparently maintain self tolerance but enhance susceptibility to infection might rely on different mechanisms is an open question of greatest clinical relevance.
